# Improving access to safe blood is critical to reducing maternal mortality in sub-Saharan Africa

**DOI:** 10.1038/s43856-025-01267-x

**Published:** 2025-11-27

**Authors:** Tunde Oyebamiji, Imodoye Abioro, Olamide Bello, Chiamaka Esther Amaefule, Oyewale Osundeyi

**Affiliations:** 1 School of Public Health, Imperial College London, London, UK; 2 Healthbotics LLC, Ibadan, Nigeria; 3 Mersey and West Lancashire Teaching Hospitals NHS Trust, Merseyside, UK; 4 Health and Wellbeing (Public Health) Department, Camden Local Health Authority, London, UK; 5 Withybush General Hospital, Haverfordwest, UK

**Keywords:** Public health, Disease prevention

## Abstract

Oyebamiji et al. highlight the high maternal mortality occurring in sub-Saharan Africa, driven in large part by inadequate access to safe blood. They examine how systems level governance and sustainable innovation can close this gap and advance maternal health equity across the region.

## Teaser

Maternal mortality remains unacceptably high in sub-Saharan Africa, driven in large part by inadequate access to safe blood, particularly in rural areas. Here, we examine how systems-level governance and sustainable innovation can close this gap and advance maternal health equity across the region.

In 2023, an estimated 260,000 women worldwide died from complications related to pregnancy and childbirth^1^. Sub-Saharan Africa (SSA) accounted for over 70% of these deaths, reflecting a severe and persistent inequity in maternal health. The World Health Organization supports the Sustainable Development Goal 3.1, which aims to reduce the global maternal mortality ratio to fewer than 70 per 100,000 live births by 2030^1^. Currently, SSA remains off-track to meet these goals.

The five leading causes of maternal mortality globally are postpartum haemorrhage (PPH), sepsis, pre-eclampsia/eclampsia, obstructed labour, and unsafe abortion^1^. Among these, PPH is the most common, and the most preventable. Each year, an estimated 14 million women suffer from PPH, leading to around 70,000 maternal deaths^2^. In SSA, PPH is estimated to account for more than 30% of all maternal deaths^1,3^, a burden slightly higher than the global average of 27%, underscoring the region’s disproportionate vulnerability^1^.

While medications and surgical interventions are important in managing PPH, blood transfusion is often the only life-saving option in cases of severe bleeding^4^. This is particularly true when blood loss exceeds 1000 mL or leads to hemodynamic instability^5^. Yet, equitable access to safe and timely blood remains a major challenge across SSA. Many countries in the region lack the regulatory frameworks, infrastructure, and sustainable financing needed to support universal access to safe blood^6^ which leaves thousands of women without a lifeline in moments of critical need.

In this comment, we explore the importance of access to safe blood for maternal health and examine the persistent barriers that hinder equitable access in SSA. We also discuss how actors within global health governance and sustainable innovations can help close this gap, accelerating progress toward the Sustainable Development Goal 3.1.

## Clinical and Public Health Value of Safe Blood

Safe blood is defined as blood collected from voluntary, non-remunerated donors that is properly tested, processed, stored, and transfused to recipients without causing harm, including avoidance of the transmission of infectious diseases^7^. The transfusion of safe blood is critical in the management of PPH, as recognized by its inclusion in the World Health Organization’s Model List of Essential Medicines and in guidelines for comprehensive emergency obstetric care.

There is a well-established inverse relationship between the availability of safe blood and maternal mortality^8^. Countries with the lowest availability of blood tend to have the highest rates of maternal death^9,10^. One modelling study in a high-income setting estimated that the absence of blood transfusion could result in a 6.5-fold increase in the risk of maternal death^10^. Although few studies in SSA have specifically quantified this effect, over half of the literature on severe PPH in the region identifies the unavailability of blood as a contributing factor^11^. In Malawi, the establishment of a national blood transfusion service led to a 50% reduction in maternal deaths within two years, indicating the impact this improvement could have on maternal mortality^12^.

Treatment options for PPH are limited in SSA. While medications such as heat-stable carbetocin and tranexamic acid, the only two drugs approved in the last 30 years for PPH, can reduce bleeding, they are often inaccessible in SSA due to distribution and storage limitations^2^. Surgical intervention is frequently required in cases of uncontrolled haemorrhage, and its success hinges on the timely availability of safe blood.

## Barriers to Universal Blood Access in Sub-Saharan Africa

Access to safe blood for women before, during, and after childbirth remains unequal between high- and low-income countries, with women in low-resource settings disproportionately affected^13^. Even within SSA, stark disparities persist as blood transfusion services are often concentrated in urban centres, leaving rural and remote communities with limited or no access to timely, safe blood^6^.

The World Health Organization’s strategy for ensuring blood safety, which is endorsed by the World Health Assembly, focuses on five key areas: nationally coordinated transfusion systems; quality-assured testing for infections and compatibility; appropriate clinical use to minimize unnecessary transfusions; end-to-end quality control systems; and voluntary, non-remunerated blood donation^8^. Yet, in many SSA countries, progress in applying these strategies is hampered by chronic underfunding, weak infrastructure, and workforce limitations. Low blood donation rates, in particular, remain a major bottleneck^14,15^.

Of the estimated 118.5 million blood donations collected globally in 2021, less than half came from developing countries, despite these countries comprising over 82% of the global population^7^. To meet population health needs, WHO recommends a minimum of 10 units per 1000 people per year^15^. SSA falls far below this benchmark, averaging just 4.9 units per 1000 people in 2022^16^.

Limited national financing remains a major constraint, with many sub-Saharan African governments allocating inadequate resources to blood transfusion services^6^. These budget shortfalls impact every component of the transfusion chain, from infrastructure and personnel to testing and logistics, ultimately compromising the quality, safety, and availability of blood and blood products, particularly for pregnant women, who are the largest group of transfusion recipients in the region^7^. Increased funding could address several of these challenges by strengthening infrastructure, expanding workforce training, and enhancing cold chain systems.

However, beyond financial constraints, several systemic barriers continue to hinder equitable access to safe blood. These include weak health governance, reliance on out-of-pocket payments, poor road networks limiting delivery, a persistent shortage of skilled healthcare workers, and fragmented supply chain coordination^14^. Addressing these barriers will require not only sustained financial investment but also policy reform, cross-sectoral collaboration, and long-term capacity building.

## The role of global health governance in making safe blood more accessible

Global health governance encompasses the collective actions of state and non-state actors, including international organizations, private sector entities and civil society groups, to shape and implement policies addressing shared health challenges^15^. Effective governance relies on credible, inclusive, and evidence-based decision-making to achieve equitable and sustainable health outcomes^17^.

In the context of maternal health and blood access in SSA, global health governance plays a pivotal role in tackling systemic barriers such as inadequate infrastructure, limited financing, and low voluntary blood donation rates^6,14^. Global actors support progress by setting strategic agendas, harmonizing national blood policies, and fostering coordination across institutions^6^. They also mobilize resources, design equitable distribution frameworks, and focus attention on underserved communities most affected by shortages^6^. Crucially, the implementation of robust monitoring and accountability systems enables ongoing evaluation of blood system performance and supports continuous improvement^6^.

The Saving Mothers, Giving Life (SMGL) initiative illustrates how governance frameworks can drive measurable impact. Using a district-level model centred on real-time data, multisectoral coordination, and accountability, SMGL improved emergency obstetric care, including blood availability^18,19^. In Zambia, maternal deaths from postpartum bleeding dropped by 65%, and facility-based deliveries rose by over 44% between 2012 and 2016^18,19^. In Uganda, both institutional and districtwide maternal mortality declined by 44% over five years in SMGL-supported districts^18,19^ demonstrating that implementation of solutions vastly improved outcomes through structured support. Although specific transfusion data remain limited, enhancements in emergency services and blood access were core components of the intervention^18,19^.

At the regional level, the West African Health Organization (WAHO) has advanced blood safety by promoting policy harmonization across ECOWAS member states^20^. In Burkina Faso, support from WAHO and partners helped increase voluntary blood donation from 37% in 2010 to over 65% in 2015, contributing to a 15% reduction in maternal deaths from haemorrhage at major hospitals^16^. In Benin, WAHO-backed reforms, which include policy alignment and decentralized blood storage, led to a rise in health districts with functional blood banks from 42% in 2015 to 76% in 2019, and a 30% decline in stockouts in rural maternity wards^21^. These examples serve as evidence that addressing both the limitations of donation and the logistics related to access can improve maternal mortality rates.

While such governance models remain underutilized in much of the Global South, they highlight the need for comprehensive approaches that move beyond increasing blood donation alone. Strengthening transfusion infrastructure, financing mechanisms, and policy implementation is essential to address the full spectrum of access barriers. This systems-wide perspective, increasingly emphasized in the access-to-medicines literature, should underpin future efforts to improve blood access and reduce maternal mortality across SSA.

## Innovating sustainable solutions to improve safe blood access

Progress towards reducing the maternal mortality ratio to fewer than 70 deaths per 100,000 live births remains unacceptably slow in SSA. With PPH posing the greatest threat to maternal survival, sustainable innovations offer an opportunity for the region to avoid traditional barriers and address the persistent rural-urban divide in access to safe blood.

Such innovations can strengthen every step of the blood supply chain, from boosting voluntary donations to accelerating emergency delivery. In Nigeria, Healthbotics LLC’s “Lend an Arm,” a digital health venture supported by the Resolution Project and the United States African Development Fund (USADF), leverages mobile technology to encourage blood donation, applies point-of-care diagnostics for faster testing, and connects hospitals to nearby blood banks in real time using an agile logistics solution^22–24^. In Rwanda, the company Zipline, in partnership with the government, uses drone delivery to overcome poor road infrastructure and ensure timely access to blood, particularly for obstetric emergencies in rural areas^25^.

Expanding financial protection is another essential component of a sustainable solution. Including blood services in national health insurance packages can significantly reduce the cost burden for patients, particularly during obstetric and emergency care^26^. Yet, in many SSA countries, blood remains excluded from insurance benefits^26,27^. This causes additional financial burdens to families, forcing them to pay themselves during life-threatening emergencies. These financial barriers leave countless women without access to timely transfusions when they are most needed.

For these innovations to be effective and equitable, they must be integrated into national health systems, appropriately regulated, and adapted to local contexts. Sustainable innovations will require not only novel technologies but also the policy frameworks and institutional capacity to scale them inclusively and alleviate barriers preventing program success.

## Conclusions

With the 2030 deadline for the Sustainable Development Goals fast approaching, SSA faces a significant challenge in reducing maternal mortality, much of which stems from preventable PPH. Blood transfusion alone could avert up to one-quarter of maternal deaths annually, yet access to safe blood remains severely limited in many countries across the region^28^. Without significant investment in blood transfusion services, most countries in SSA are unlikely to achieve the SDG target for reducing maternal mortality.

Universal access to safe blood must be addressed through a whole-systems approach that considers the broader determinants and persistent barriers, including voluntary blood donation, sustainable financing, safety assurance, delivery and removal of financial burdens to patients through insurance changes. Achieving this will require coordinated action across global health governance actors to develop integrated, equity-driven solutions and create policy environments that support their effective implementation. Sustainable innovations, when effectively regulated and embedded within national systems, can also help the region bypass entrenched access gaps and bring safe blood within reach for all who need it, whilst advancing maternal health equity in the process.
